# Acute Gastroenteritis and Campylobacteriosis in Swiss Primary Care: The Viewpoint of General Practitioners

**DOI:** 10.1371/journal.pone.0161650

**Published:** 2016-09-07

**Authors:** Philipp J. Bless, Joan Muela Ribera, Claudia Schmutz, Andreas Zeller, Daniel Mäusezahl

**Affiliations:** 1 Swiss Tropical and Public Health Institute, Basel, Switzerland; 2 University of Basel, Basel, Switzerland; 3 Partners for Applied Social Sciences (PASS) Suisse, Neuchâtel, Switzerland; 4 Centre for Primary Health Care, University of Basel, Basel, Switzerland; Universita degli Studi di Parma, ITALY

## Abstract

Acute gastroenteritis (AG) is frequently caused by infectious intestinal diseases (IID) including food- and waterborne pathogens of public health importance. Among these pathogens, *Campylobacter* spp. plays a major role. Many European countries monitor selected IIDs within disease surveillance systems. In Switzerland, the information on IIDs is restricted to limited surveillance data, while no data is available for AG. We conducted a qualitative study among Swiss general practitioners (GPs) to investigate the case management of AG and campylobacteriosis patients, the associated disease burden and the determinants leading to registration in the National Notification System for Infectious Diseases (NNSID). Interviews were conducted with a semi-structured questionnaire and underwent inductive content analysis based on Grounded Theory. The questionnaire was repeatedly adapted to capture emerging themes until the point of theoretical saturation. GPs perceived AG and campylobacteriosis of little relevance to their daily work and public health in general. According to GP self-estimates each consults about two cases of AG per week and diagnoses a median of five campylobacteriosis cases per year. A large proportion of AG cases receives telephone consultations only and gets medical advice from the practice nurse. Antibiotic therapy is considered useful and stool diagnostics are performed for about a fifth of consulting AG patients. Stool diagnostics (“test”) and antibiotic therapy (“treat”) are interrelated and follow four strategies: “Wait & See”, “Treat & See”, “Treat & Test”, and “Test & See”. AG case management is diverse and includes different triage steps. A small proportion of AG patients have stool diagnostics performed and only positive tested patients are reported to the NNSID. As a result severe cases and cases with a history of travel abroad are overrepresented in the NNSID. The use of multiplex PCR panels in routine diagnostics likely leads to improved case management and higher case numbers in surveillance systems.

## Introduction

Acute gastroenteritis (AG) is characterised by diarrhoea (watery, bloody), vomiting, fever, abdominal pain and cramps, nausea, and dehydration that occur in different combinations and with varying degrees of severity [1–3]. Those suffering from AG are frequently affected by infectious intestinal diseases (IID) caused by a wide range of gastrointestinal pathogens like viruses, bacteria and other parasites [1, 4, 5]. Food- and waterborne pathogens such as *Campylobacter* spp. and *Salmonella* spp., for example, are of particular public health concern as they can lead to disease outbreaks in addition to causing sporadic cases [6–8]. For this reason, many IID causing pathogens are monitored in most European Union (EU) countries and in Switzerland [9, 10]. The Swiss National Notification System for Infectious Diseases (NNSID) monitors a range of food- and waterborne pathogens including *Campylobacter* spp., *Salmonella* spp., *Shigella* spp. and enterohaemorrhagic *Escherichia coli* [9]. Among these, the most frequently notified IID in Switzerland is campylobacteriosis. Since 2006, a dramatic increase in case notifications has been observed, with an all-time high of almost 8,500 notified cases in 2012 [11]. Increasing trends in case notifications were also observed in the European Union (EU) [10] e.g. England and Wales [12] or Germany [13] and in the United States of America (US) [14]. The NNSID is the only source of routine information on IIDs among the Swiss population, but it does not cover syndromic surveillance of AG, nor does any other surveillance system.

The overall aim of the NNSID is to allow for the early detection of disease outbreaks and health threats from infectious diseases to initiate timely interventions for disease control. Additionally, the system supports a continuous assessment of existing preventive measures. Only laboratory-confirmed cases of notifiable IIDs are reported to the NNSID. Reported case data include the patient’s personal data (name or initials, address or place of residence, sex, age), the applied diagnostics, the diagnosing laboratory and the physician in charge [9]. However, except for enterohaemorrhagic *E*. *coli*, the NNSID does not collect associated clinical information such as onset of disease, signs and symptoms, progression of disease, case management, hospitalisations, risk exposures or risk factors for IIDs [9]. In addition to insufficient knowledge on the clinical presentation of IIDs, the actual burden of IIDs and AG at the primary care level and the population level are unknown. To assess the disease burden from laboratory-based surveillance data at both levels, it is crucial to know the patients’ health care seeking behaviour and the physicians’ case management including diagnostic practices. The lack of such information considerably impedes ability of the NNSID to capture minor epidemiological trends and interpretation of its data. The aims of this qualitative study among Swiss general practitioners (GPs), were to investigate the case management of AG and campylobacteriosis patients, to assess the influence of patient’s health care seeking behaviour and of GPs’ clinical decision making on surveillance data and to collect estimates on the incidence of AG and campylobacteriosis at the primary care level.

## Materials and Methods

### Questionnaire development

We developed a semi-structured questionnaire for face-to-face interviews that was informed by the study objectives, expert opinions and relevant literature. The questionnaire was divided into two parts. The first part covered *GPs’ perception of AG and campylobacteriosis;* that is, the perceived magnitude of the burden of AG and campylobacteriosis, incl. semi-quantitative estimates, relevance to public health, the clinical presentation (signs and symptoms) in daily practice, patients’ health care seeking behaviour (motives and processes), and the patients’ profile as it relates to risk behaviours and risk groups. The second part, *Case management*, focused on the case management of AG and campylobacteriosis by evaluating diagnostic practices and treatment approaches (incl. influencing factors and logic behind the action) and reasons for related decisions, like referral to a specialist or hospitalisation.

### Interviewer training and pilot testing of questionnaire

Pilot and study interviews were conducted by three female social scientists (SF, MZ and SH) and one male epidemiologist (PJB), between May and August 2013. The interviewers received multiple trainings in qualitative interviewing techniques from a senior medical anthropologist (JMR). Pilot testing of the questionnaire consisted of a preliminary interview with a key informant (senior GP), followed by five test interviews in German (four) and French (one). After the pilot, the questionnaire was re-structured accommodating the common procedure during the medical consultation with a patient with AG. The pilot indicated that the variety of determinants and approaches for symptomatic treatment are rather limited. Therefore, the questionnaire rather focused on examining the complex determinants and approaches for antibiotic therapy.

### Recruitment of GPs and interview procedure

GPs who had managed campylobacteriosis patients in a previous case-control study [15] but were otherwise not actively engaged, were invited for an interview for the purpose of the current study. In addition to those 146 German-speaking and 29 French-speaking GPs of the case-control study [15] we purposely recruited six French-speaking GPs for the study to better represent the French-speaking area of Switzerland. GPs were invited with an information letter sent by postal mail. After the anticipated receipt of the information letter, GPs were contacted by telephone and the study and study objectives were described. Verbal informed consent was obtained and an appointment for the interview arranged. The interview was conducted at a place of the GP’s choice, which was usually in his or her own practice. Interviews generally lasted for 20–40 minutes and were tape recorded and transcribed.

### Data analysis

Data analysis followed the principles of inductive content analysis as required by Grounded Theory and was performed with Weft-QDA software (http://pressure.to/qda/). Upon completion, interviews were immediately transcribed and iteratively analysed, while data collection was ongoing. This approach allowed us (i) to capture emerging themes that could be included in subsequent interviews, (ii) to refine the question guide and (iii) to evaluate the saturation process. Codes for data analysis were continuously developed and assigned to GPs’ narratives. All interviews were coded by a senior medical anthropologist (JMR). Theoretical saturation of themes and factors was eventually reached and study results were discussed at length by the research team. Semi-quantitative estimates of the perceived magnitude of AG and campylobacteriosis and the rates for requesting faecal specimens are given as the reported median and range.

### Ethics statement

The work presented in this article and the previous case-control study [15] formed a project mandated by the Swiss Federal Government studying the epidemic increase of human *Campylobacter* spp. infections in Switzerland. Over the last decade notification rates for campylobacteriosis had not only steadily increased between 2005 and 2012 but also weekly notification rates peaked annually at the turn of the year. In 2011/2012 weekly notifications increased extraordinarily twofold compared to the previous and following weeks [11]. In concert with the general epidemiological trend this situation was categorised as an epidemic threat by the Federal Government. In response the Federal Office of Public Health (FOPH) commissioned the project for the winter 2012/2013 enforcing the Swiss Epidemics Act (SR 818.101 EpG). Projects conducted under the Epidemics Act do not require ethical approval. Hence, we did not seek approval from an ethical committee for the study but conducted the study in line with the Declaration of Helsinki. Participating GPs provided verbal informed consent. They received an information letter of the FOPH and were subsequently contacted by telephone. During the telephone conversation interviewers explained again the purpose of the study and repeated the content of the information letter. GP’s were subsequently formally asked to participate and their response check marked on the consent form. We did not obtain written informed consent as the interviews focused solely on GPs’ professional views about the subject matter and not on any personal aspects or data of individual patients. The GPs’ personal data were anonymised and they did not receive any financial compensation for their participation.

## Results

### Characteristics of participating GPs

In total, 69 GPs participated in the study (51 German-speaking and 18 French-speaking). The participation rate among GPs from the previous case-control study was 36.0% (63/175). Of the study participants, 13 (18.8%) were female and 56 (81.2%) were male. The majority (62) of interviewed GPs had specialised in general internal medicine, while five specialised in paediatrics, one in anaesthesia and one in urology. The latter two also provided primary health care. The median professional experience of GPs was 23 years (range: 3–39 years) and the median number of patients consulted per GP per week (as estimated by the GPs) was 138 (range: 32–300). Slightly more than half of the interviewed GPs (38/69) worked at a practice located in a semi-urban community, and practices located in urban and rural communities accounted for 30.4% (21/69) and 14.5% (10/69) of the sample.

### Perception of acute gastroenteritis and campylobacteriosis

Nearly all interviewed GPs considered AG in Switzerland to be of little relevance for the patient, uncommon in daily practice and of minor public health importance in Switzerland (Table 1). In contrast, GPs highlighted that AG plays an important role in travel medicine and patients with a travel history. Interviewed GPs estimated observing a median of 2 cases of AG per week (range: 0–10 cases per week) and a median of 5 (range: 0–52) laboratory-confirmed cases of campylobacteriosis each year. GPs highlighted that the real number of campylobacteriosis cases is higher than that indicated by laboratory-confirmed cases due to patients’ health care seeking behaviour (not all AG patients contact a GP) and GPs’ testing behaviour (not all AG cases are tested). The general perception was that, *Campylobacter* spp. has surpassed *Salmonella* spp. as the primary cause of bacterial diarrhoea in Switzerland, compared to the 1990s. Campylobacteriosis cases occur in waves or phases throughout the year and usually peak during the summer months and between December and January. GPs explicitly linked the summer peak to barbequing and the winter peak to traditional consumption of meat fondue (Table 1).

**Table 1:** Perception and burden of acute gastroenteritis and campylobacteriosis in Swiss primary care.

Participant	Quotes
SFY12	*“I have the impression that they [campylobacteriosis and AG] are not such a public health problem*. *Because I do*, *if I have them*, *if I discover them*.* *.* *.*I treat them*. *I don’t have the impression that they…in any case for me…they are not a problem for me*.*”*
MZ20	*“(…) Diarrheal diseases only become problematic when there is an electrolyte and fluid imbalance*. *Most at risk are children*. *(…) But all in all*, *it is not a problem*. *Diarrheal diseases are generally self-limiting*.*”*
MZ20	*“So*, *first of all*, *I do not diagnose every campylobacter case or every bacterial diarrhoeal case*. *I only conduct targeted testing*. *I do not do routine testing in the case of diarrhoea*. *This means that I certainly miss some of the cases*.*”*
SF02	*“Indeed*. *Before*, *the main problem was Salmonella*. *I have rarely seen Shigella*. *Very rarely*. *Nowadays*, *campylobacter is more common*.*”*
SF01	*“In summer*, *it can be [observed] during the barbeque season*, *it [barbeque] is a fostering element*.*”*
MZ01	*“One can see this after every festivity day*. *So after Christmas*, *people show up with campylobacter infections*. *This is due to the poultry*. *Fondue chinoise [meat fondue] is stretched out with poultry*.*”*
	*“After the fondue chinoise season there are increasing campylobacter infections*, *yes*.*”*
PJB16	*“Mostly it is fondue chinoise or barbeques*. *That is the classic*. *Nowadays it is rarely eggs or sauces compared to earlier times*, *but fondue chinoise is really classic*.

GPs agreed on the basic signs of bacterial AG, particularly for campylobacteriosis: symptoms like abdominal pains and cramps or fever appear abruptly and the patient feels and presents as very ill (Table 2). Nausea and vomiting were also mentioned but occur less frequently. Some GPs also mentioned pain in the limbs and headache. Campylobacteriosis was seen as a self-limiting disease, easy to treat, and generally not dangerous for peoples’ health (Table 1). However it can lead to a severe, painful and disturbing health condition that prevents people from working (Table 2). GPs recognised the importance of AG and campylobacteriosis for vulnerable patients such as infants, the elderly, or individuals suffering from co-morbidities. Campylobacteriosis can affect anyone, independently of age, sex or socio-economic status. However, young adults and middle-aged people appear to be affected more frequently than the rest of the population, and especially more frequently than vulnerable groups. The perceived risk factors for contracting campylobacteriosis mentioned were: handling and eating raw or undercooked poultry, travelling and eating “unsafe” food, eating out in canteens or restaurants, barbecuing, consuming meat fondue with poultry or ready-to-eat salads and working in the food sector. Campylobacteriosis patients are generally unable to work for several days to more than one week (Table 2). The patient’s general condition is the main criterion for sickness certification and duration of sick leave. Other medical factors linked to occupation (physical or nonphysical activities; activities that can put others at risk) or social factors (like pressure by the employer or the patients themselves) also play a role.

**Table 2:** Clinical presentation and risk groups for campylobacteriosis.

Participant	Quotes
PJB17	*“It’s [symptoms of campylobacteriosis] for sure relatively fast appearing diarrhoea*, *watery diarrhoea*, *nausea*, *frequently fever*. *So they are really doing badly for a few days*.*”*
MZ13	*“They [campylobacteriosis cases] mostly have fever*. *But it is mostly not very high*. *Whereby this also occurs for viral infections*.* *.* *.* *.*The patients have also a bad general condition*. *Blood [in stool] I don't see so often*. *(…) The patients simply feel bad*. *When one only has a gastro-intestinal flu*, *one doesn't feel fit*. *One also has to run to the toilet all the time*. *But somehow*, *people with campylobacter really look very bad*. *(…)They are very pale and almost collapse*.*”*
MZ01	*“Campylobacteriosis goes across all the social strata*, *all generations*, *across everything*.*”*
PJB21	*“No*, *it [the risk group] is less children*, *mostly age groups from 20–25 to 60–65 years*, *this middle group*. *Less so children and older people*.*”*
PJB16	*“Until they [the patients] start to improve a little after three to four days*, *until they are healthier again it goes approximately one week*. *Then they return to work*, *except if they have a physical work*, *then*, *it might need a little bit longer*. *But people working in the office or students can go back to work after a week–still a little impaired*, *not completely normal yet*. *Then it gradually improves*.*”*
PJB14	*“Independent of the stool test*, *the patient is anyway ill for three to five days and can’t go for work*. *For patients working in the food sector*, *maybe even ten to fourteen days*. *The employers don’t really like it because it is a long time*.*”*
PJB05	*“Another reason [for treating with antibiotics] is the importance of the working position of the patient*. *Some really need to go to work*, *others do not*. *Some ask for it [antibiotics]*.*”*

### Health care seeking behaviour and medical encounters at the primary care level

GPs reported on patients’ individual health care seeking behaviour. Individuals affected by AG consult their GP within several hours to days after the onset of symptoms. Factors accounting for prompt or delayed patient consultation included perceived severity, pain and distress, past experiences, attitude towards coping with disease, health insurance deductible or the need for a medical certificate. Up to 60% of all AG-related enquiries lead to telephone consultations only, without a face-to-face consultation at the practice. Thus, practice nurses play a key role in evaluating the severity of disease, filtering patients for consultations at the practice and providing appropriate medical advice on the telephone. Several physical (e.g. severity), psychosocial (e.g. anxiety, mutual trust), and situational (e.g. GPs’ workloads) factors can favour either telephone or face-to-face consultations. After the first consultation and with appropriate measures taken, most GPs either schedule a follow-up appointment (usually by phone but sometimes at the medical practice) or ask patients to call if the symptoms do not improve. The follow-up serves as a means for evaluating the course of disease and for establishing further actions if needed. GPs’ workloads can be an obstacle to routine follow-up. Medical treatment is either concluded passively, i.e. patients do not contact the GP again, or actively at a follow-up consultation.

### Diagnostic and treatment approaches

Routine consultation of an AG patient starts with history taking, including assessment of potential risk exposures followed by a clinical examination and point-of-care diagnostics (e.g. C-reactive protein (CRP) level). Faecal specimens for diagnostic purposes (mainly stool-cultures for *Campylobacter* spp., *Salmonella* spp. and *Shigella* spp.) are requested for a median of 18% (5–60%) of AG patients depending on the general condition, fever, blood in faeces, elevated CRP level e.g. >100 mg/l, prolonged disease duration, relevant co-morbidities, patient’s occupation and a positive history of ingesting risky food or of travel (Table 3).

**Table 3:** Diagnostic approaches for AG cases among Swiss GPs.

Participant	Quotes
MZ23	*“For each patient*, *I first make an anamnesis*. *I ask him since when has he had it*. *When did it start*? *Have you eaten something special*? *Have you done something special*? *Have you been abroad*? *Just the anamnesis*. *After this*, *the first impression of the patient*. *When there is massive diarrhoea*, *you can see that the patient is suffering*. *An important symptom is fever*. *Febrile diarrhoea has to be looked at differently*. *(…) Then I usually take CRP and blood status*. *Harmless diarrhoea has mostly a CRP of 20 to 40*. *But campylobacter have often 100*, *120*. *Sometimes*, *they also have a leucocytosis*. *And when I have a suspicion*, *I request a stool examination*.*”*
MZ19	*“Fever*, *bad general condition and when the patient himself says that he feels bad*. *Then I do a blood test*, *so CRP and leucocytes*. *I only check stool bacteriology when the values are clearly increased*.*”*
PJB06	*“An anamnesis revealing a risk situation*. *I say it like this*, *did he have a risk situation*, *did he eat eggs*, *or poultry products or mozzarella or such products somewhere*, *so farmer products (…) or does he have fever*?*”*
MZ16	*“In fact*, *there are two reasons for which one generally makes a stool examination*: *If the patient really feels ill and miserable*. *(…) As a general rule*, *I then give Imodium*. *When the patient has taken it correctly*, *and it doesn't work*, *then I might do a stool test*. *Then there is a second group*: *Patients who were abroad*. *For these patients*, *it is possible that I primarily do a stool test*. *(…) Then there is a third group*, *where I primarily do a stool examination*. *When the patients come from an old people's home and one knows that there are already several people who fell ill*.*”*

Symptomatic treatment of AG, including antimotility drugs and oral rehydration therapy for simple cases or intravenous rehydration therapy for severe cases, is very common (Table 4). Antibiotic therapy plays only a secondary role due to the self-limiting nature of most AG cases. Nevertheless, antibiotic therapy is considered useful but prescribed cautiously. Its indication depends on disease severity, general condition, fever, inflammation parameters, occupation and partially on stool diagnostic results (Table 5). GPs mostly prescribe ciprofloxacin and to a lesser extent erythromycin or specific classes of antibiotics, depending on the stool diagnostic result. Most GPs were concerned about potential antibiotic resistance of gastrointestinal bacteria. However, only some remembered experiencing this problem in their medical practice (Table 5). GPs were aware that frequent prescription of antibiotics is positively associated with the occurrence of antibiotic resistance. However, many also consider antibiotic therapies as very helpful for individual treatment, even if not medically indicated, to shorten disease duration or to ameliorate symptoms, for example.

**Table 4:** Symptomatic treatment approaches for AG cases among Swiss GPs.

Participant	Quotes
MZ11	*“So primarily*, *I administer probiotics*. *At first*, *I focus on nutritional establishment and [recommend] the intake of fluids with light meals and without any dairy products for two*, *three days*. *Like this*, *one manages the patient slowly*. *This is standard for me*.*”*
PJB18	*“There is the “solution of thirds”*. *(…) one third orange juice*, *one third black tea and one third mineral water*, *heavily sugared*. *(…) It contains everything*, *potassium in the orange juice*, *bicarbonate in the mineral water and you have fluid and sugar*. *(…) [it] is a cheap electrolyte solution*.*”*
PJB02	*“And then only if…someone has 12 stools per day*, *I also give antidiarrhoeals*. *This is something that relieves the symptoms*. *But otherwise*, *in the first 3 days*, *cleaning of the intestine belongs to the body’s self-healing processes*.*"*
PJB06	*“So what I mean*, *what I sometimes do*, *I give someone an infusion*. *When somebody is prone to collapsing and has low blood pressure with nothing risky otherwise*. *Then we do an infusion here or at home*. *This I indeed like to do*, *I like to offer this*.*”*
PJB21	*“They do essentially get better and the fever decreases faster if one gives an infusion and puts the fluid balance a little back in order*.*”*

**Table 5:** Prescription of antibiotic therapy for AG cases and the perception of antibiotic resistance.

Participant	Quotes
MZ23	*“If somebody feels really sick and has a high fever*, *I may give him antibiotics quicker than recommended*. *(…) But on the other hand*.* *.* *.*if somebody has a fever of 39 degrees for two days and diarrhoea*, *you don't leave him to wait for another two*, *three days*. *With these cases*, *I am relatively easy in giving antibiotics*.*”*
PJB17	*“The indication of antibiotics is generally not due to the test result campylobacter*, *but rather due to the symptomatology”*
MZ18	*Interviewer*: *“When do you give antibiotics*?*”*
	*“If there is extremely high fever and there are extremely high inflammation values*, *a CRP of 100 or higher*.*”*
PJB14	*“If I don’t know the pathogen yet*, *I empirically give ciprofloxacin*.*”*
	*Interviewer*: *“And after you get the test result*?*”*
	*“If it is Campylobacter jejuni*, *I change to a macrolide”*
MZ15	*“In fact*, *I almost always treat with ciprofloxacin*. *I attended a tropical course*. *There I have heard that resistances are building slowly and that erythromycin products would be better*. *But up to now*, *I have always had good experiences with ciprofloxacin*.*”*
PJB02	*“The resistance problem is known*. *Often there is a quinolone-resistance and then macrolides have to be used*. *It is only a matter of time until resistances also appear there*.*”*
PJB18	*“Yes I had one single case that did not react to it [ciprofloxacin]…with campylobacter*. *There*, *I had to do a second culture with an antibiotic resistance profile*. *Then it worked*, *indeed with azithromycin and not with ciprofloxacin*.*”*

### The interplay of stool diagnostics and antibiotic therapy

Initiating stool diagnostics (“Test”) is interrelated with antibiotic therapy (“Treat”) and follows four distinct approaches to acting and reacting in specific medical, social and physical situations (Table 6). GPs can lean towards “Treat & See”, “Treat & Test” or “Test & See”, and some can “Wait & See” longer than others. Few respondents openly refused an individual approach or adhered to one of these approaches only. The approaches “Wait & See” and “Treat & Test” appeared to be preferred.

**Table 6:** Diagnostic and antibiotic therapy approaches among Swiss GPs.

Approach	Quotes
**Wait & See**	PJB08: *“Waiting doesn’t mean omitting*. *Watchful waiting as it is called*. *It is a pleasant fact that*, *in general*, *a lot of problems that we are confronted with in general medicine are self-limiting*. *For this reason*, *I do not have to make a big effort concerning diagnostics*. *One decides based on the evidence (observing the course of the disease) and says*: *‘Come again in two days’*. *Mostly they have to come again for a medical certificate*. *Or I tell the patient to report within a certain period if it doesn’t improve*.*”*
	MZ18: *“Basically*, *one goes ahead step by step*. *First*, *one observes*. *One waits*. *One leaves it open*. *One only does a stool culture when diarrhoea persists*. *Not for every patient*.*”*
	Interviewer: *“So the self-healing tendency plays a role*?*”*
	MZ18: *“Exactly*. *One waits*, *often for one week or so*. *But when the pains are extreme or it takes longer*, *then we take a stool culture*.*”*
**Treat & See**	MZ17: *“I am convinced that for 99% of the cases*, *we don't know the disease agent*. *We treat with a broad spectrum antibiotic*. *I cannot make a throat smear for every patient with sore throat (…)*. *I think this is not the objective of a general practitioner*.*”*
	Interviewer: *“So in the end you treat blindly*. *So*, *independent of a test result*.*”*
	MZ18: *“Yes*, *exactly*. *This is in our science not unusual*. *One applies a broad spectrum antibiotic*. *(…) For a lung infection*, *for example*, *you of course do not know whether a patient has this or that bacterium*. *This is of no interest to me in the general practice*. *And in general*, *it does not help the patient either*. *If you make a culture then you have the result maybe next Wednesday or Thursday*. *When you must send the sample you have to consider that the patient has pains and inflammation values for the next four*, *five days*. *What is the examination good for*? *It costs the patient money*. *In the deductible-system the patient pays*, *without any use*.*”*
**Treat & Test**	MZ03: *“And then there is such a thing*.* *.* *. *there is such a cookbook rule*, *if there is bloody diarrhoea*, *or febrile diarrhoea*. *After doing stool bacteriology I give mostly ciprofloxacin*. *I say*: *“You go home first*, *then you fill the container*, *and then you swallow these tablets”*. *And this has shown quite good results*. *(…) And I say he should not take the tablets beforehand*, *otherwise we do not know what it is and we have no diagnosis*, *if it does not get better*. *(…) And I know it [test result] within four days*. *“Usually you [the patient] get better*. *But if you do not feel better*, *we know which bug it is*.*"*
	MZ15: *“Mostly*, *one also does a CRP*, *in order to determine whether it is a bacterium or a virus*. *(…) Depending on the result of the blood examination*, *I give him [the patient] a tube to take home*. *I always say then that it costs a lot of money*. *If you feel better*, *we don't need to send it*. *One has to treat anyway before one has the result*. *(…) it is practically always blind treatment*. *It is rare that the laboratory calls and we have not yet treated*. *It is then rather a confirmation that something is there*. *I*, *in fact*, *almost always treat with ciprofloxacin*. *(…) When I have the impression that the patient does not necessarily want to know it*, *I take the stool sample and I start treatment*. *When he gets better*, *we don't send it*.*”*
	MZ19: *“When a patient comes with this psycho-social pressure*, *for example he has to work*, *then you do give him antibiotics*. *Then I don't wait until I receive the results*. *I treat immediately*.*”*
**Test & See**	PJB03: *“When the patient says he hasn’t improved at all*, *the test result is there*, *maybe against expectation…of campylobacter*, *and he says*: *‘I don’t get better at all’*, *he has still stomach cramps*, *diarrhoea and so on*, *then I treat him”*
	Interviewer: *“So it might take some time until you get the test result*. *What is the influence of this in your treatment*?*”*
	PJB23: *“Generally none*. *In the first phase*, *the treatment is independent of the stool sample*.*”*
	Interviewer: *“In the first phase*. *What do you do then with a positive result*?*”*
	PJB23: *“Let’s say it is salmonella or campylobacter positive*, *it doesn’t mean that they get ciprofloxacin*. *It simply means that one keeps an eye on them*. *I look how fast they recover and follow-up after a week*, *checking inflammation indicators*.*”*
	Interviewer: *“So a positive test result doesn’t always indicate an antibiotic use or so*?*”*
	PJB23: *“No*. *(…) A young healthy person can overcome these diseases without antibiotics*, *they are not necessary*. *Second*, *there is a risk that we will have more chronic carriers if we give antibiotics*. *For salmonella this is known*, *they recover faster but they remain longer carriers*, *really for a long time and this is what I want to avoid*.*”*

#### Wait & See

This approach seems to be the standard starting point for most AG episodes. It is based on the principle that symptoms of AG including campylobacteriosis disappear after two to five days. It is mostly applied when the episode is recent and mild, or if the patient is in good general condition. Practice nurses evaluate the patient by telephone and decide if there is a need for a consultation or if the patient should wait out the disease’s progression. Diet, rehydration and symptomatic treatment recommendations are provided at this stage.

#### Treat & See

Few GPs reported treating AG with antibiotics without requesting a faecal specimen. The underlying logic is that there is no need to know the exact cause of AG for a successful treatment, particularly if there is indication of a bacterial infection, such as an elevated CRP level. Other reasons were: the costs of stool diagnostics, wish to reduce the duration of suffering and infeasibility of requesting a faecal specimen (if the patient has to travel or the episode occurs just before the weekend, for example). The approach is a pragmatic one, focused on patients’ wellbeing and against the perceived norm for cautious use of antibiotics.

#### Treat & Test

Antibiotic therapy starts before knowing the stool diagnostic result but after faecal specimen collection. It can then be modified upon receiving diagnostic results. This approach implies that empirical treatment usually works and that stool diagnostics are helpful for the post-diagnostic adaptation of antibiotic therapy. GPs’ responses indicate the need to start antibiotic therapy immediately due to social (e.g. the patient has to work) and medical considerations (e.g. bad general condition). Reasons for applying this approach include the possibility of redirecting treatment if indicated, and considering public health aspects (e.g. if the patient works in the food sector or in health care).

#### Test & See

This approach implies that antibiotic therapy only starts if indicated and after knowing the stool diagnostic result. However, antibiotics are only indicated if bacterial pathogens are identified and symptoms persist or the patient’s general condition deteriorates. Then the approach transforms to “Test and Treat” and the patient receives the pathogen-specific antibiotic therapy. GP’s applying this approach seek to avoid unnecessary and empirical ‘best-guess’ antibiotic therapies.

### Referrals

Generally, GPs manage AG patients themselves at their practices. Complex cases of AG are referred to a specialist (gastroenterologist, specialist for infectious diseases, specialist in tropical and travel medicine) or a hospital. Reasons for referring a patient to a specialist include the development of persistent or chronic gastroenteritis (e.g. diarrhoea persists several weeks, prolonged blood in faeces) or no response to the usual treatments. A specialist in travel medicine is specifically approached if gastrointestinal problems persist after travelling abroad. Hospitalisation of AG patients is rather uncommon. Hospital referrals occur in case of bad general condition, severe dehydration, fear of sepsis, suspicion of diverticulitis or appendicitis or if vulnerable patients suffer from severe AG. They also occur in cases where patients cannot manage at home due to lack of social support (e.g. elderly people living alone) or travel (e.g. tourists staying in a hotel).

## Discussion

A qualitative study among 69 GPs in Switzerland on the clinical presentation and case management of acute gastroenteritis and campylobacteriosis showed that GPs see around two patients with AG per week and a median of 5 campylobacteriosis cases per year. However, AG patients can also treat themselves at home, sometimes with medical advice from the practice nurse. Campylobacteriosis and AG are perceived as having little relevance for general public health, daily clinical practice and the average patient. Case management in the form of antibiotic therapy and stool diagnostics follows four approaches: “Wait & See”, “Treat & See”, “Treat & Test” or “Test & See”. GPs request faecal specimens for stool diagnostics from 18% of AG patients and prefer empirical antibiotic therapy before stool diagnostic results are available over result-based antibiotic therapy.

### The burden of acute gastroenteritis and campylobacteriosis

GPs generally observe that, among causes of IIDs, *Campylobacter* spp. has surpassed *Salmonella* spp. in the last 20 years. This is confirmed by the trends of campylobacteriosis and salmonellosis case numbers reported to the NNSID [11]. Similar trends in notification rates were observed in the EU [10] e.g. Wales [16] whereas in the US the incidence of *Salmonella* spp. remained higher than for *Campylobacter* spp. [14, 17]. NNSID data also support GPs’ impressions that young adults and middle-aged people are more frequently affected [11]. More prevalent exposure risks among these groups, such as traveling abroad, eating out and preparing food themselves, as stated by GPs, could be responsible for increased case numbers. However, data to support this assumption are not yet available for Switzerland.

The described seasonality of campylobacteriosis cases is reflected in the NNSID data [11]. Two distinct peaks of campylobacteriosis, one during summer months and one shorter peak over the festive season in December and January, lead to more primary care attendance. The summer peak of campylobacteriosis case notifications is observable throughout Europe [10]. The winter peak has been described in detail for Switzerland and Germany and is also observable in European notification data [10, 11, 13]. GPs associated the frequent consumption of meat fondue over the festive season in winter with increased campylobacteriosis case numbers. Indeed, meat fondue consumption was found to be the major risk factor for the winter peak of campylobacteriosis in Switzerland, as it is tradition to consume it at Christmas and New Year times [15]. The association of the summer peak with the barbeque season is plausible as barbequing meat provides many occasions for undercooking and re- and cross-contamination [18]. Studies in Switzerland [19] and Germany [20] showed higher *Campylobacter* spp. contamination rates of chicken meat during summer months. Additional drivers for infection could also be more frequent recreational water activities or travels in summer, both risk factors that have been previously described for campylobacteriosis [18, 21–23]. GPs also observed that consultations due to AG occur in a clustered manner, with alternating case-free weeks and then several cases occurring in a single week. This might be due to small, local epidemics of viral IIDs. Switzerland does not have routine syndromic surveillance of AG, which would allow investigations of temporal and seasonal AG trends. This would be desirable, as other European countries such as France have had positive experiences with routine syndromic surveillance of AG [24].

### The influence of patients’ health care seeking behaviour on NNSID case numbers

Many AG affected individuals do not contact a GP at all or only get advice by telephone, depending on their health care seeking behaviour. GPs are aware that this leads to an underestimation of the IID burden at the primary care level and has—together with case management approaches—an influence on NNSID case numbers. This has already been described for other disease surveillance systems [5, 25, 26]. However, patients suffering from a bacterial gastrointestinal infection appear to be more likely to consult a GP than patients with a viral gastrointestinal infection [5, 27]. In the Netherlands, national GP guidelines recommend telephone consultations by practice nurses to deal with simple AG cases to reduce the number of consultations and stool diagnostics [28]. In Switzerland, the active promotion of telephone consultations for patients with mild AG could help to reduce health care expenditures, which are among the highest in the world [29]. According to study GPs, severely affected patients often directly consult the emergency department of a hospital, whereas the average AG case is dealt with at the practice and is rarely referred to a specialist or hospital. This is comparable with other findings reporting 8.5% of GPs hospitalising an AG patient during the seven days preceding the interview [30]. However, hospitalised AG patients suffering from an IID are likely to undergo intensive diagnostics and, hence, will not be missed by the NNSID if diagnosed with a notifiable disease.

### The influence of diagnostic approaches on NNSID case numbers

The GPs’ self-estimated proportion of requesting faecal specimens for 18% of patients is comparable to other studies where rates vary between 4.3% and 50% [25, 28, 31–33]. Individual rates differed strongly among the GPs interviewed. The observed heterogeneity seems to be rather common and has also been observed among English GPs [31]. It is likely related to GPs’ individually perceived usefulness of stool diagnostic results for case management and the patient populations they serve. This highlights the need to systematically estimate the faecal specimen testing rate to assess the disease burden of notifiable IIDs at the primary care level from NNSID case numbers.

The determinants for requesting a faecal specimen, as found by this study, are similar to those found in other studies [28, 30–32, 34–36] and are consistent with published recommendations on the clinical management of AG cases [4, 37]. Additionally, our study showed that factors related to the health system (e.g. health insurance deductible or duration of stool diagnostics) also influence the decision to perform stool diagnostics. An important determinant for performing stool diagnostics was the patient’s CRP level. An elevated CRP level is considered indicative of a bacterial infection, making distinct stool diagnostics more likely. Arguments for this criterion were the limited treatment possibilities for viral causes of AG and the need to know the bacterial cause for targeted antibiotic therapy. Requesting a faecal specimen based on a positive travel history, as observed in our study, may not always be appropriate as stool diagnostics are not recommended for watery or traveller’s diarrhoea due to the low yield of recognising pathogenic bacteria (e.g. enterotoxigenic *E*. *coli*) in the sample [37]. In accordance with others [32, 34], we observed that mainly severely affected patients or patients with a history of travelling abroad undergo stool diagnostics in Switzerland, likely leading to a high proportion of severe and imported cases in the NNSID. Imported and domestic cases cannot be distinguished in the NNSID for most IIDs as laboratory reports do not include any information on exposure. Hence, the possible overrepresentation of imported cases should be considered when interpreting NNSID data as they are of less relevance for assessing national disease transmission and interventions. To improve the interpretation of NNSID data it would be advisable to include patients’ recent travel history on case notifications to differentiate between imported and domestic cases, similarly to other European countries [10]. The preference of severe cases for stool diagnostics also explains the perceived high severity of disease by notified cases (7 on a rating scale from 1 = not severe, to 10 = very severe) and the high antibiotic prescription rate (61.6%) found in our case-control study on determinants of campylobacteriosis in Switzerland [15].

When the study was conducted, the first diagnostic laboratories had introduced multiplex polymerase chain reaction (PCR) panels for IIDs in routine diagnostics. Up until then, the routine stool diagnostics applied to AG patients were stool-cultures for *Campylobacter* spp., *Salmonella* spp. and *Shigella* spp. [38]. Only a few of the interviewed GPs had already deliberately ordered stool diagnostics with this new diagnostic tool. Multiplex PCR panels will likely affect case numbers in the NNSID if they are routinely deployed by Swiss GPs. They have a higher detection rate of IIDs in faecal specimens compared to conventional methods due to a higher sensitivity and the wide range of IIDs tested simultaneously [39–41]. Greater sensitivity will likely lead to increased case numbers of the routinely tested and notifiable IIDs (*Campylobacter* spp., *Salmonella* spp. and *Shigella* spp.) within the NNSID. Similarly, stool diagnostics for other specific notifiable IIDs, e.g. enterohaemorrhagic *E*. *coli*, were mainly requested for AG patients with a certain suspicion such as blood in faeces. More tests will be conducted for these IIDs because faecal specimens investigated by multiplex PCR panels are tested for the same range of IIDs independent of the suspected cause which could lead to the detection of more cases.

### “Treat & See” and “Treat & Test” for targeted antibiotic therapies

The GPs in our study considered stool diagnostics and antibiotic therapy useful for managing AG cases. The “Wait & See” approach, including symptomatic treatment, is the approach applied most often to AG case management among Swiss GPs. This is in line with published case management guidelines for simple AG cases [1, 4, 37, 38]. From a public health perspective, the “Treat & See” and “Treat & Test” approaches are questionable as both can lead to untargeted antibiotic therapies. Similar to the “Wait & See” approach, the “Treat & See” approach additionally contributes to the underreporting of IID cases in the NNSID.

Studies have shown that a large proportion of faecal specimens from AG patients do not identify viral pathogens and, hence, disease is likely not caused by bacteria [42–45]. The aforementioned approaches bear a high probability of incorrectly treating those patients with antibiotics. In the era of increasing antibiotic resistance among gastrointestinal bacterial pathogens, untargeted antibiotic therapy should be avoided. Additionally, antibiotic therapy needs to be carefully considered for its potentially counter-productive effects for bacterial infections such as *Escherichia coli* O157:H7, for example [46, 47]. Timely antibiotic therapy is desirable to reduce disease duration and to increase the wellbeing of the patient in cases of severe AG (e.g. with febrile dysentery with an indication of a bacterial cause such as an elevated CRP level or based on food history) [4, 36, 37].

A major reason for applying the “Treat & See” and “Treat & Test” approaches is the perceived long duration until culture-based stool diagnostic results are available. Therefore, fast molecular diagnostics for IIDs such as multiplex PCR panels would enable the physician to initiate timely and targeted antibiotic therapy and are desirable [36, 39]. When these are widely deployed, “Test & See” could become the preferred approach to AG case management over “Wait & See”. The fast availability of diagnostic results will also permit a shift to “Test and Treat”, including a specific and timely treatment approach based on stool diagnostic results. GPs in our study were prone to change their treatment approach based on the stool diagnostic results. However, immediate antibiotic therapy will remain the therapy of choice for severely affected patients to assure the wellbeing of the patient.

Swiss surveillance data shows that *Campylobacter* spp. is the most frequent bacterial cause of IIDs [11]. But around 50% of tested *Campylobacter* spp. isolates from humans are resistant to fluoroquinolones according to Swiss study and surveillance data [48, 49]. Therefore, the prescription of ciprofloxacin (fluoroquinolone) for AG cases with a suspected bacterial cause, as mentioned by interviewed GPs, is questionable. Azithromycin (macrolide) would be the drug of choice for treating campylobacteriosis and is also appropriate for treating salmonellosis and shigellosis [4, 37]. A similar level of resistance of *Campylobacter* spp. to fluoroquinolones is observed in EU countries, but varies considerably between countries. As a result, the European Food Safety Authority and the European Centre for Disease Prevention and Control no longer consider fluoroquinolones appropriate for routine empirical treatment of human campylobacteriosis [50]. In summary, empirical antibiotic therapy for the treatment of AG patients should be avoided whenever possible and macrolides (e.g. azithromycin or erythromycin) are recommended for empirical treatment if it is indicated for the wellbeing of the patient.

### Discussion of research approach

A wide range of GPs was accessible through the previously conducted case-control study [15] and provided an ideal and unique opportunity to assess the case management of AG and campylobacteriosis patients and the associated disease burden at the primary care level. A qualitative research approach was chosen due to the lack of information on AG and campylobacteriosis at the primary care level in Switzerland and the unknown willingness of GPs to participate. This allowed researchers to collect information on all aspects of interest nearly independent of the participation rate, but limited the possibilities for quantifying some of the results. Semi-quantitative estimates on the disease burden by GPs allowed a first assessment of the unknown disease burden at the primary care level. Such estimates of disease burden should be interpreted with caution as they are influenced by several factors. The progression of the interview, the time point of the interview in regard to disease seasonality or the GP’s importance alluded to the disease can lead to over- or underestimation. The large sample of 69 GPs from the German- and French-speaking parts of Switzerland increased the geographical and paradigmatic variation represented, leading to an improved saturation of investigated themes. Additionally, interviewers followed-up on various topics with different levels of detail during the interviews due to different backgrounds of interviewers, resulting in even wider variation and fast theoretical saturation. We lack interviews with GPs from the Italian-speaking part of Switzerland but we assume that–due to the minor differences between French- and German-speaking GPs–differences in case management and disease burden would only slightly differ from our study results. It might appear that the study generated only general knowledge, but it is the first and largest study to date providing a comprehensive overview of the applied case management, disease burden and determinants leading to disease notification in Switzerland.

## Conclusions

The health care seeking behaviour of AG patients leading to primary care attendance, and GPs’ varying case management approaches including triage steps and stool diagnostic frequency need to be taken into account when interpreting NNSID data. Patients severely affected by AG or who travelled abroad are more frequently seeking care and are hence, overrepresented among campylobacteriosis cases notified in the NNSID. As a result, the NNSID monitors the epidemiological situation of notifiable IIDs of more severe disease expressions rather than the entire spectrum of notifiable IID suffering in the Swiss population. The current transition from routine culture-based stool diagnostics to routine multiplex PCR panels in diagnostic laboratories will likely counter act such a skewed epidemiological data situation. This expectation is mainly driven by the higher sensitivity of these molecular diagnostics and by a possible increase in the number of stool diagnostics conducted due to faster availability of diagnostic results. Therefore, the anticipated increase in case numbers will not necessarily reflect an epidemiological trend in the Swiss population and should be considered when communicating NNSID data to stakeholders. Knowledge on which diagnostic methods are available and actually applied is important for public health authorities to accurately interpret NNSID data, particularly during the transition period. Consequently, further research should be conducted on the impact of routine multiplex PCR panels on the composition and number of cases registered in the NNSID and possible changes in case management including antibiotic therapies.
